# Engineering of luminescent graphene quantum dot-gold (GQD-Au) hybrid nanoparticles for functional applications

**DOI:** 10.1016/j.mex.2020.100963

**Published:** 2020-06-17

**Authors:** Shikha Wadhwa, Alishba T. John, Ashish Mathur, Manika Khanuja, Gourav Bhattacharya, Susanta S. Roy, Sekhar C. Ray

**Affiliations:** a Amity Institute of Nanotechnology, Amity University Uttar Pradesh, Noida 201313, India; b Centre for Nanoscience and Nanotechnology, Jamia Millia Islamia, New Delhi110025, India; c Shiv Nadar University, Dadri, Gautam Budhha Nagar, Uttar Pradesh 201314, India; d Department of Physics (CSET), University of South Africa, Private Bag X6, Florida Science Campus, Florida -1710, Christiaan de Wet and Pioneer Avenue, Johannesburg, South Africa

**Keywords:** GQD-Au, Reduction of metal salts and organic dyes, Wastewater treatment

## Abstract

Graphene quantum dots (GQDs) possess excellent optical and electrical properties that can be used in a wide variety of application. Synthesis of hybrid nanoparticles with GQDs have been known to improve the properties further. Therefore, in this method, graphene quantum dots –gold (GQD-Au) hybrid nanoparticles were synthesized using GQDs which reduces HAuCl_4._3H_2_O to Au nanoparticles on its surface at room temperature. The GQDs with self-passivated layers were synthesized by microwave assisted hydrothermal method using glucose as a single precursor. The synthesis process does not involve the use of harmful chemicals. The whole synthesis process of GQD and GQD-Au hybrid nanoparticles takes only five minutes. The synthesized GQDs have been extracted using citrate in order to increase the stability of the hybrid nanoparticles for up to four weeks. The size of the synthesized GQD-Au hybrid nanoparticles is in the range of 5–100 nm and were found to be luminescent under UV-A illumination. The merit of the following method over other synthesis techniques include its rapidity, ease of preparation, and no requirement of elaborate synthesis procedures and/or harmful chemicals. The GQD-Au hybrid nanoparticles can be used in several applications such as luminescent coatings for glass and windowpanes for automobiles, etc. The reducing property of GQDs can further be utilized for the reduction of various metal salts (AgNO_3_) and organic dyes (methylene blue and methyl orange).

. It presents a method/protocol-development of the luminescent GQD-Au hybrid particles of size ~ 5–100 nm.

. The GQD-Au hybrid particles find potential applications in luminescent coating applications.

## Graphical abstract

**Figure:**
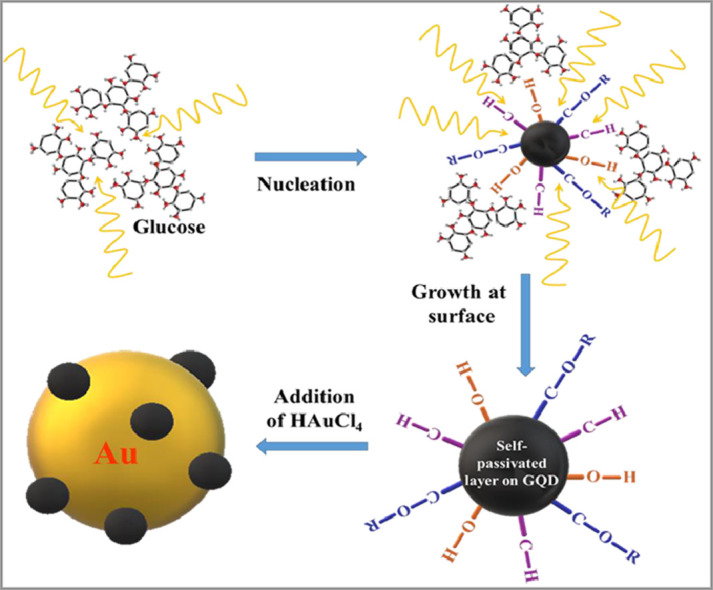

Specification Table

**Table:** 

Subject area:	Environmental and biological science
More specific subject area:	Luminescent coatings and waste water treatment
Method name:	A facile room temperature microwave assisted hydrothermal method
Name and reference of original method:	R. Liu, D. Wu, X. Feng, K. Müllen, Bottom-up fabrication of photoluminescent graphene quantum dots with uniform morphology. *J. Am. Chem. Soc.***133** (2011) 15,221–15,223.

This is a direct submission

## Introduction

### Background

The concept of quantum dots has been making rounds in the scientific community since 1980; nevertheless, it was only after a decade that researchers were able to synthesize successfully size tunable QDs [1,2]. One of the most commonly investigated QDs were CdX QDs (*X* = *S*, Se, Te), due to its excellent electrochemical and optical properties, but, with the increase in biological application, toxicity of CdX has become a major concern. In order to solve the problem of cytotoxicity with the use of CdX based QDs, researchers have been exploring the properties of other cadmium-free QDs such as carbon-based QDs, silicon QDs, metal nanoclusters owing to their excellent biocompatibility and luminescent properties.

Carbon based QDs such as carbon dots (C-dots) and graphene QDs (GQDs) has been making the buzz due to their excellent optical and electrical properties. Scientists have been recently attracted towards GQDs mainly because of its tunable bandgap properties in addition to pronounced quantum confinement, good electron mobility, chemical stability and edge effects leading to its application in multiple arenas [3], [4], [5]. Depending on the technique employed for its preparation, reports have concluded that GQDs can have a size ranging between 1 and 60 nm that synthesized at higher temperature [6]. GQDs exhibit various environmental and biological applications such as optical sensors, detection of biomolecules and bioimaging [7], photocatalysis and photoelectrocatalysis [8] and removal of organic dyes by adsorption [9]. GQD-metal hybrid particles demonstrate wide applications such as detection of biomolecules [10], visible light photocatalysis [11], optical imaging, photothermal effect and thermometry [12], and therapeutics [13]. Hence, GQD-metal hybrid particles represent functional nanomaterials for wide range of applications.

Herein, we report for the first time, a room temperature synthesis of GQD-Au hybrid nanoparticles via reduction of Gold (III) chloride trihydrate (HAuCl_4__•_3H_2_O) by preformed GQDs. Self-passivated GQDs were synthesized by microwave-assisted hydrothermal method which involves pyrolysis of glucose.

### Application of the method

This facile methodology maybe employed for the reduction of other metal ions from their stable salts to metal atoms (such as Ag, Ni), thereby resulting in the synthesis of GQD-metal hybrid nanomaterials (see Anticipated Results).

### Comparison with other methods

Numerous top-down and bottoms-up approaches utilized for the synthesis of GQDs include laser ablation, exfoliation, hydrothermal, electrochemical oxidation, pyrolysis, microwave-assisted hydrothermal, catalyzed cage-opening, *etc.* [14]. Microwave heating or microwave assisted hydrothermal method offers numerous advantages over other synthesis techniques such as formation of uniform particle size, facile and rapid synthesis, minimal use of chemicals, easy scalability, no requirement of additional surface passivating agents, etc. Tang et al. reported for the first time, microwave synthesis of luminescent self-passivated GQDs with an average particle diameter of 3.4 ± 0.5 nm using glucose as carbon source [15]. Self-passivation layers on GQD are due to oxygen containing functional groups on its surface. The versatility of this technique included the use of carbohydrates containing C, H, and O in the ratio of 1:2:1 as starting material for synthesis of GQD as long as H and O are in the form of carbonyl, carboxyl or hydroxyl groups that eventually dehydrates under various hydrothermal conditions. The merit of using the proposed protocol for the formation of hybrid material is that, the high photonic energy possessed by microwave rays aid in the excitation of electrons present in GQDs that promotes decoupling of HAuCl_4__•_3H_2_O into radicals such as H^+^, Cl^-^ and Au^3+^. The resulting homogenous solution of GQD-Au hybrid nanomaterial is formed because of the higher reduction potential of Au^3+^ in comparison to GQDs [16,17].

### Development of the methods

GQDs were previously synthesized by our research group via pyrolysis of citric acid [18]. The drawback of the procedure included the extensive reaction conditions and use of elaborate chemicals. In order to form hybrid with other materials, for specific applications, we were interested in techniques that are quick, easily scalable and involve less chemicals. Therefore, herein, we report a room temperature synthesis of GQD-Au hybrid nanoparticles via reduction of Gold (III) chloride trihydrate (HAuCl_4__•_3H_2_O) by GQDs. The GQDs were synthesized by microwave-assisted hydrothermal method using a single precursor, glucose, without involving any harmful chemical in the process. The reduction of gold salt could not be achieved by glucose alone implying that the reducing ability of synthesized GQDs is higher than glucose for reduction of metal salts (see anticipated results).

## Method details

### Method design

**Fig. 1:**
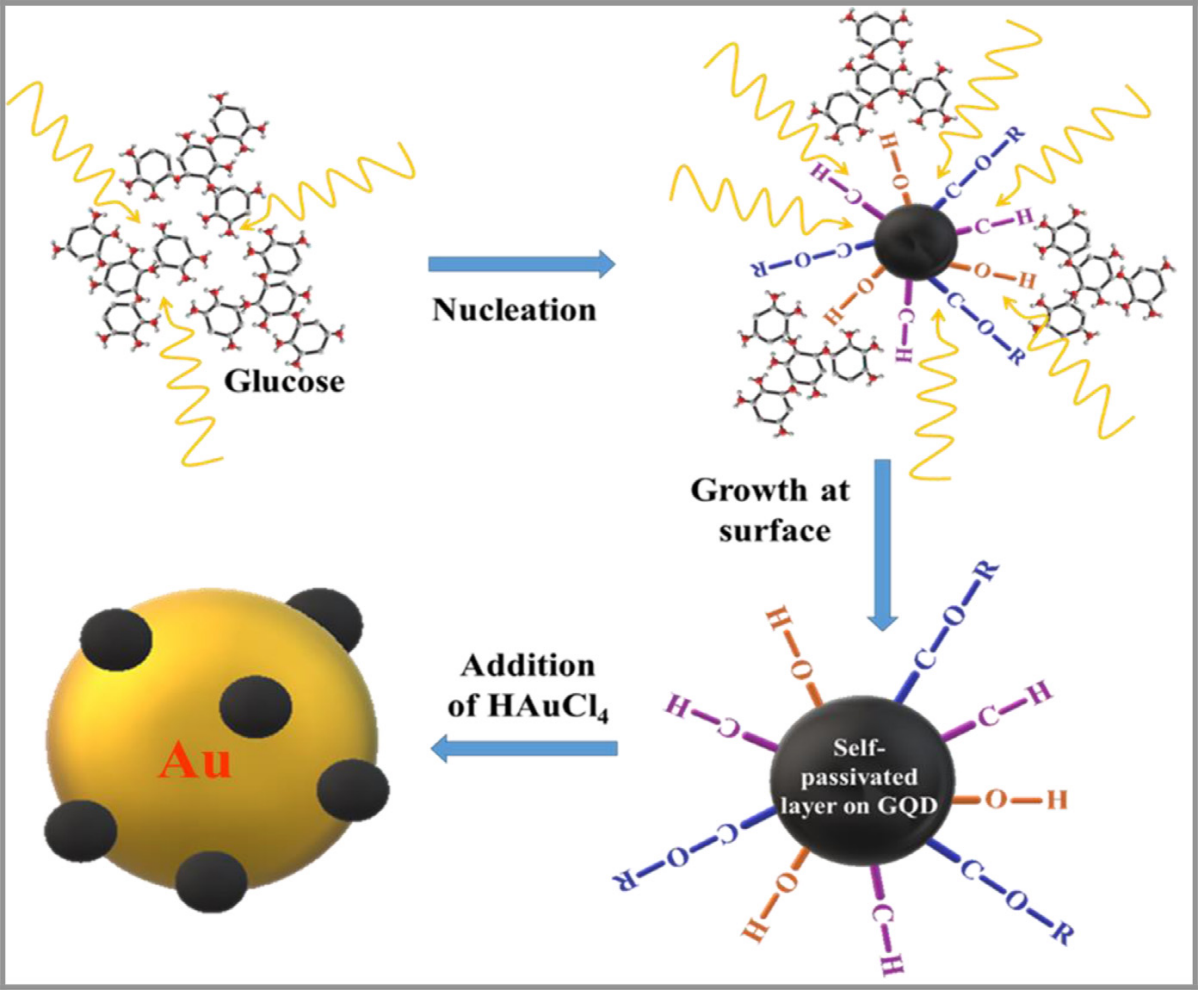
Schematic for synthesis of GQD-Au hybrid nanoparticle.

The protocol is composed of two parts: (a) microwave-assisted hydrothermal synthesis of GQD (Step 1–4) and (b) synthesis of GQD-Au hybrid particles via reduction of HAuCl_4__•_3H_2_O using GQDs (Step 5). GQDs were synthesized using glucose as a source of carbon and irradiated using a domestic microwave oven at different reaction power in the range 100 - 800 W and time in the range 2–12 min and extracted with 5 mL de-ionized water and stored at 4°C. For synthesis of GQD-Au hybrid particles, the yellow paste of GQDs obtained after microwave treatment was extracted using 5 mL 0.01 M stabilizing agent solution and stored attemperature < 4°C. Various experimental parameters such as concentration of HAuCl_4__•_3H_2_O (6–10 mM), volume of HAuCl_4__•_3H_2_O (10–40 µL), pH conditions (2–14) and effect of stabilising agent (tri-sodium citrate, PEG) were explored for the synthesis optimization of GQD-Au hybrid nanoparticles. (Fig. 1)

#### (a) *Microwave-assisted hydrothermal synthesis of GQD*

The synthesis of GQD involves pyrolysis of glucose facilitated by microwave assisted hydrothermal method. Therefore, power of microwave and reaction time are the important parameters affecting the synthesis method. Henceforth, the effect of reaction parameters is discussed below.

**Fig. 2:**
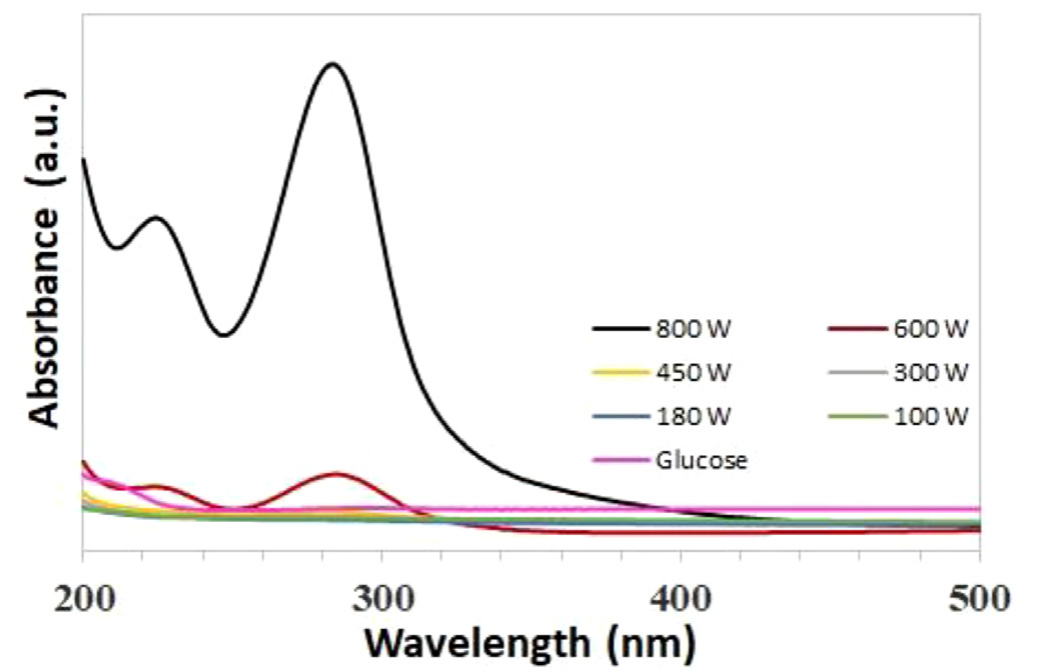
Effect of microwave power (100 W – 800 W) on the synthesis of GQD.

**(i) *Effect of microwave power*.** Glucose solutions (5 mL of 11% aqueous Glucose solution) were irradiated at different microwave power in the range 100 W – 800 W resulted in self-passivated GQDs with two characteristic absorption peaks in the UV–Vis absorption spectra. The two characteristic peaks at 225 and 286 nm indicated the synthesis of GQD (Fig. 2). Tang et al. reported characteristic absorption peaks at 228 and 282 nm and attributed to graphene quantum dots obtained by pyrolysis of glucose by microwave-assisted hydrothermal method [15]. The increase in microwave power results in increase in absorption peak intensity accompanied by redshift of absorbance band edge, whereas no change in the peak positions was observed (Fig.2).

**Fig. 3:**
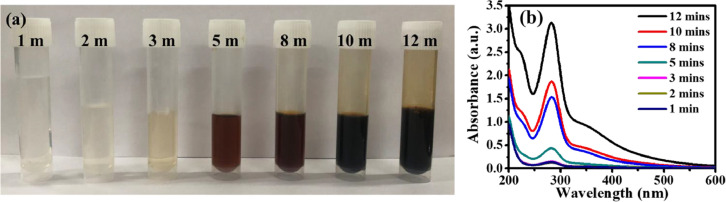
(a) Optical images showing the effect of microwave time on the synthesis of GQD extracted with water (b) UV–Vis absorption spectra of synthesized GQD at different heating times.

**Fig. 4:**
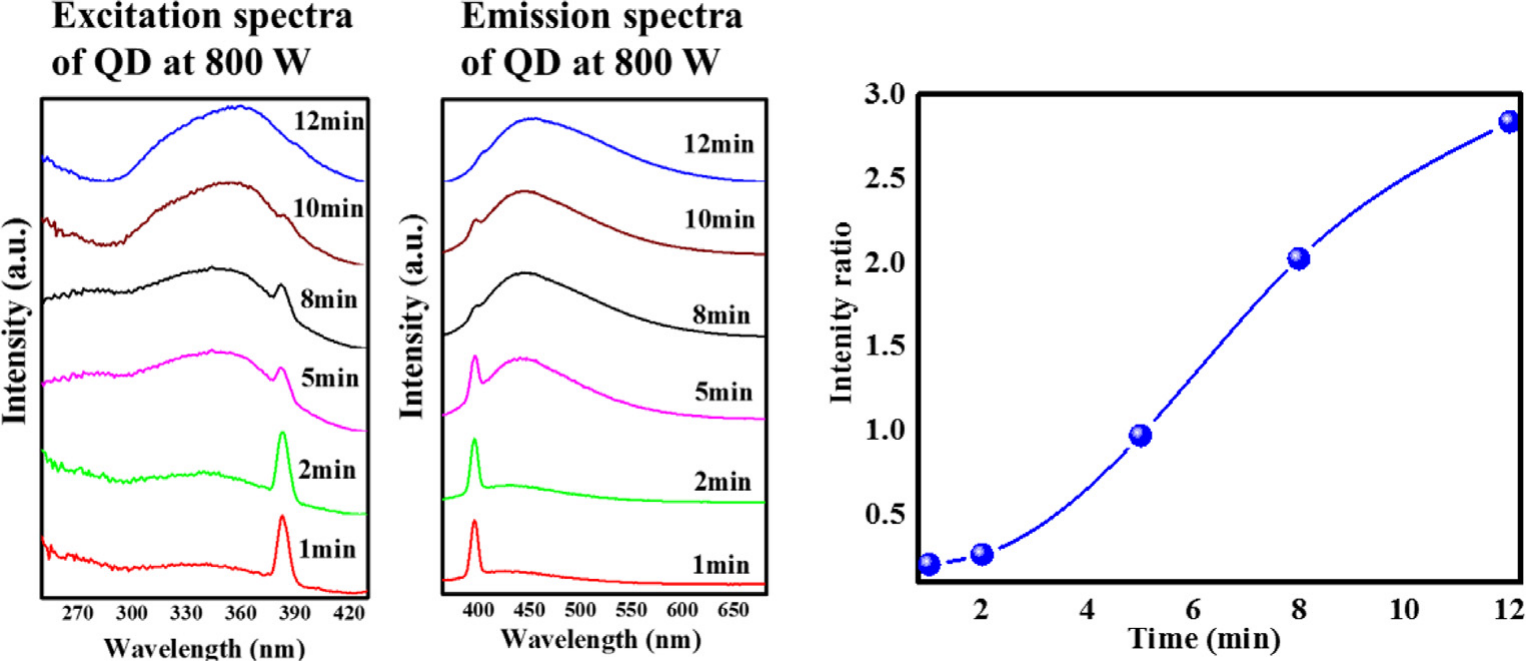
PL spectra of GQD synthesized at different time & microwave power of 800 W.

**(ii) *Effect of microwave time*.** At an optimum microwave power of 800 W, glucose solution was heated in microwave with heating time ranging from 1 to 12 min. With increase in reaction time, the colour of GQDs obtained varied from pale yellow to dark brown. Tang et al. reported that pale yellow solution contains GQDs with 10 nm diameter while dark brown solution consists of high density GQDs in the diameter range 10–21 nm (Fig. 3) [15]. Deep UV absorption intensity was found to increase with increase in heating time. It was further noted that absorption band edge exhibited red-shift while no change in peak position was observed upon increasing heating time (Fig. 3b). Fig. 4 shows the photoluminescence (PL) spectra of GQD solution at 800 W microwave power. The emission spectra collected at 350 nm resulted in two peaks around 396 nm and 443 nm. There is no change in the peak position, however only a slight variation in the peak intensity is observed. With increase in microwave time, the peak intensity increases significantly due to the transition around 443 nm. The peaks originated from the equi-coupling of π*–π and n– π induced transitions resulting in dual mode cyan emission at excitation 365 nm (~396 nm) and band-to-band π*–π *–p transition at an excitation of 429 nm (~443 nm) [19].

It was concluded that microwave power of 800 W and heating time 3–5 min are suitable to obtain GQDs with the unique optical properties for various potential applications.

#### (b) *Synthesis of GQD-Au hybrid particles*

GQD-Au hybrid particles were formed by addition of aqueous HAuCl_4__•_3H_2_O solution to the pre-formed GQD solution at room temperature. The GQDs were obtained by microwave assisted hydrothermal treatment of aqueous glucose solution for 3 min at 800 W. The resultant sticky yellow paste of GQDs obtained was extracted with 5 mL 0.01 M stabilizing agent solution prior to addition of aqueous HAuCl_4__•_3H_2_O solution. The final GQD-Au solution was stored at temperature < 4°C. Figure S1 shows the DLS of synthesized GQD-Au particles in the size range of 10–100 nm (See Supporting Information). Figure S2 demonstrates the presence of oxygen containing functional groups on GQD and GQD-Au surface (see supporting information). Following are the experimental parameters studied for the formation of hybrid nanoparticles.

**Fig. 5:**
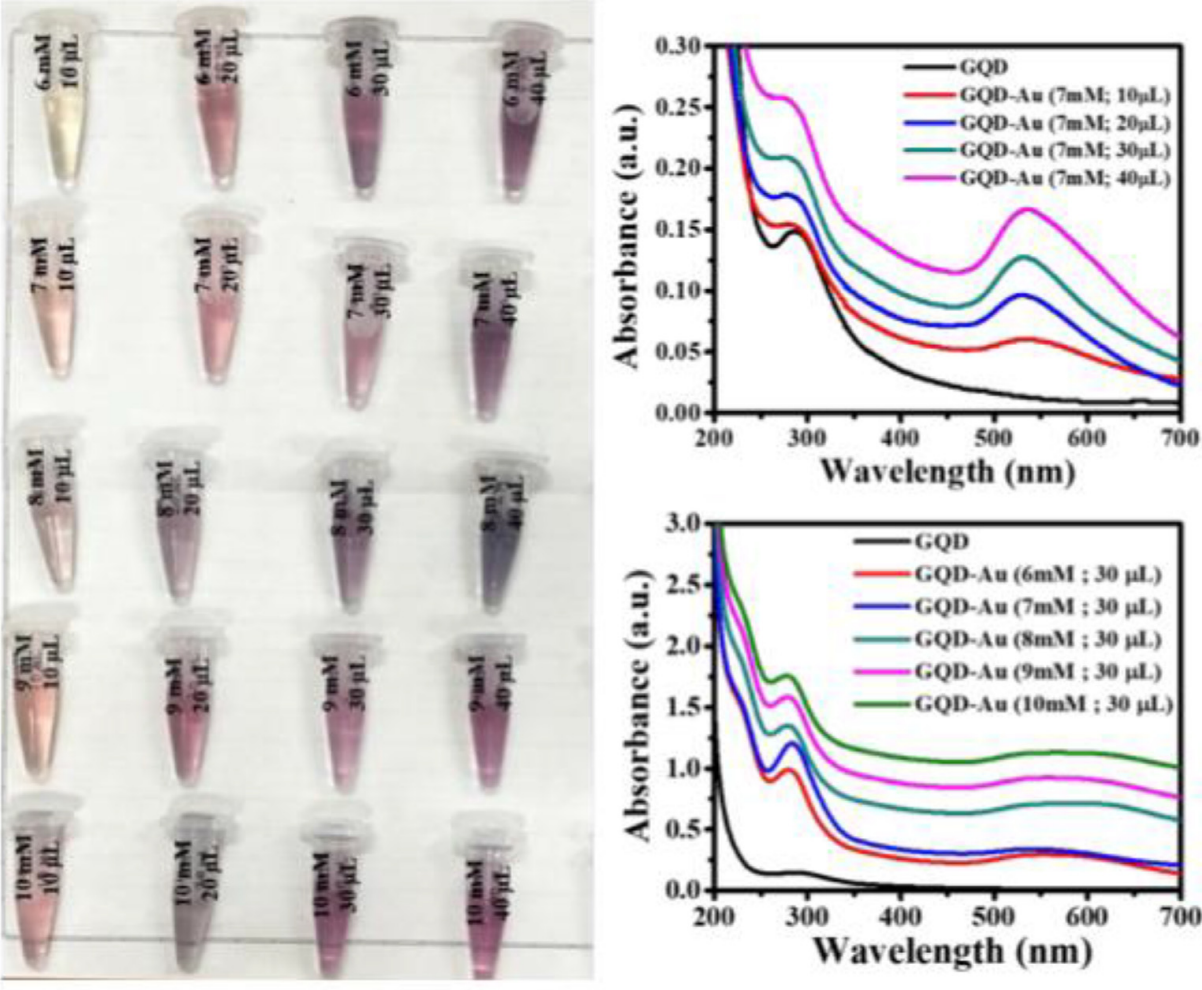
UV–Vis absorbance spectra of GQD-Au hybrid nanoparticles obtained with different (A) volumes (7 mM HAuCl_4__•_3H_2_O solution) and (B) concentrations (30 µL of HAuCl_4__•_3H_2_O).
**(ii) *Effect of* pH *on the synthesis of hybrid nanoparticles*.** Fig. 6 shows the effect of pH on the synthesis of GQD-Au. pH plays a very crucial role in the synthesis of GQD-Au hybrid nanoparticles. As the pH is varied from 2 to 12, a range of GQD-Au colloidal solutions were obtained. GQD-Au hybrid particles of smaller size were synthesized successfully at pH = 6–8 as indicated by cherry red colour of the colloidal solution. However, under highly basic conditions (pH > 8), the colloidal solution turns green due to formation of larger particles. Seol et al. obtained uniform synthesis of colloidal Au nanopartilces at pH > 6 [20] and emphasized that pH of the solution influences the species of Au solute complexes and electrostatic interaction between Au nanoparticles via transformation of AuCl_3_(OH)^-^ species into less reactive and lower redox potential complex AuCl_2_(OH)^2−^ or AuCl(OH)^3−^ at pH > 6.2. Formation of less reactive Au solute complexes favors stable reactions resulting into formation of uniform colloidal AuNPs [22]. The passivation layer on GQD, on the other hand, becomes negatively charged due to deprotonation of –OH and –COOH groups on its surface at pH > 6 enhancing its reduction potential [20].

**(i) *Effect of concentration and volume of HAuCl_4__•_3H_2_O*.** Changing the concentration and volume of HAuCl_4__•_3H_2_O strongly affects the particle size and optical properties of GQD-Au hybrid nanoparticles. Fig. 5 shows the effect of volume and concentration of HAuCl_4__•_3H_2_O on the UV–Vis absorbance characteristics of the obtained GQD-Au hybrid particles. The GQD-Au hybrid particles absorb at 282 nm and 536 nm while GQDs show absorption peak at 286 nm. The absorption intensity of both the peaks increase with increasing concentration of HAuCl_4__•_3H_2_O which is expected due to increase in the concentration of GQD-Au hybrid particles in solution. A broad absorption peak at 536 nm is attributed to Au nanoparticles. Seol et al. demonstrated microwave synthesis of Au nanoparticles of size ~ 12 nm [20]. Ngo et al. reported the size of Au nanoparticles in the range of 14–18 nm demonstrating absorption peaks in the range ~ 518–542 nm [21].

**Fig. 6:**
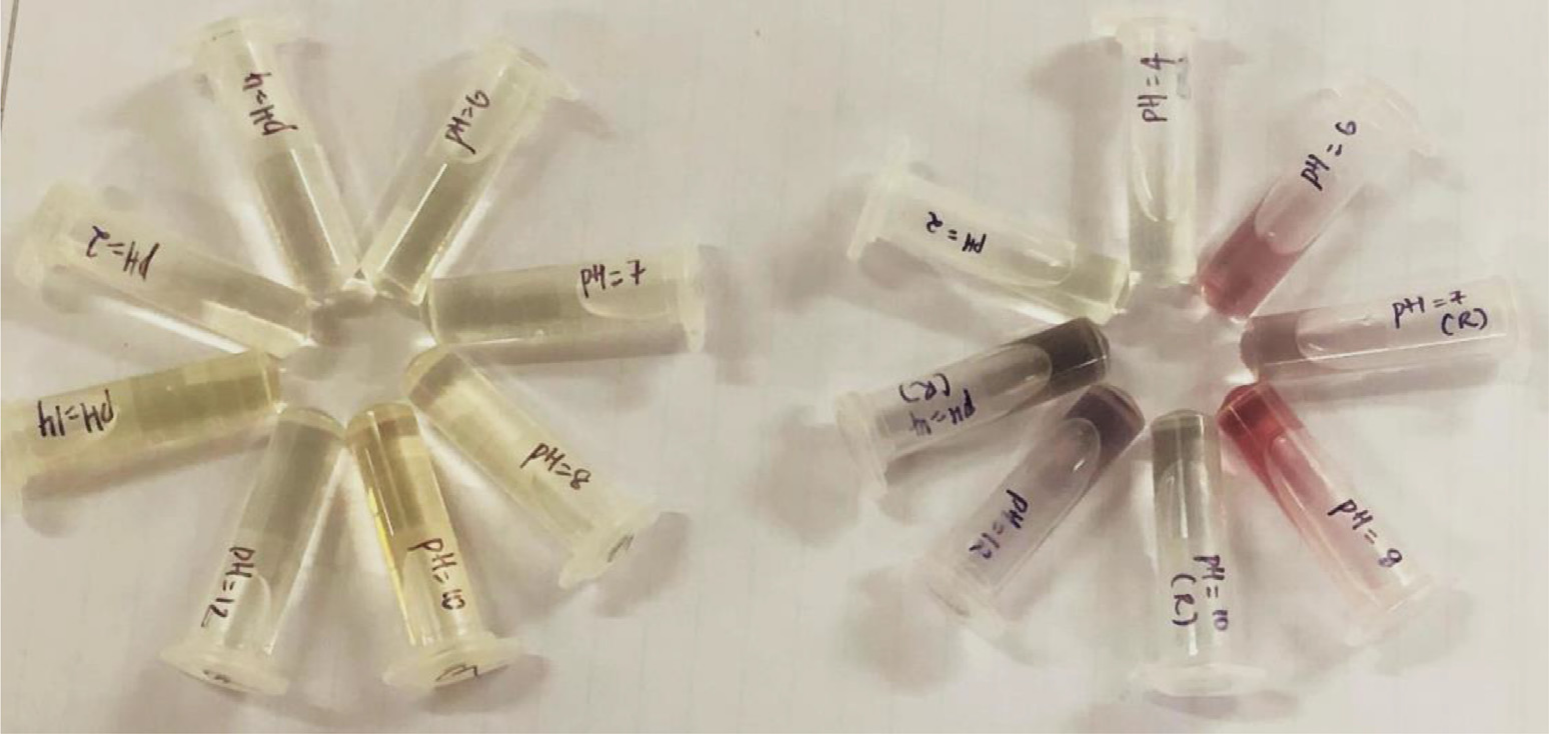
Effect of pH on GQDs (left) and GQD-Au hybrid particles (right).
**(iii) *Effect of stabilizing agent on the synthesis of hybrid nanoparticles*.** The use of water extracted GQDs for the reduction of HAuCl_4__•_3H_2_O results in the formation of larger particle size of Au as evident from blue colour of the colloidal solution (Fig. 7a). Extraction of GQD using stabilizing agents leads to particle size reduction of Au and stability of the colloidal solution. Therefore, GQDs were extracted by tri-sodium citrate or PEG (5 mL 0.01 M solution) prior to addition of Au salt precursor. Fig. 7 exhibits the effect of stabilizing agent on GQD-Au after 1 week of synthesis. The use of stabilizing agents leads to smaller particle size of Au nanoparticles over GQD surface, as evident from cherry red colour of the solution, and enhanced the stability of the colloidal solution containing GQD-Au hybrid particles upto 7 days at temperature < 4 °C. Extraction with citrate results in hybrid nanoparticles that are more stable (upto 3 weeks), in comparison to PEG. It is anticipated that the surface stabilization of GQDs affects the reducing power of GQDs due to the formation of an ester bond between the functional groups present on GQDs and that on stabilizing agents (citrate or PEG in this case). Tri-sodium citrate, being a bulkier molecule, also provides steric effects, further reducing the chemical reactivity of GQDs (Fig. 8). Consequently, surface passivation results in slow and more controlled reduction of HAuCl_4__•_3H_2_O leading to the formation of smaller Au particles and stable colloidal solutions.

**Fig. 7:**
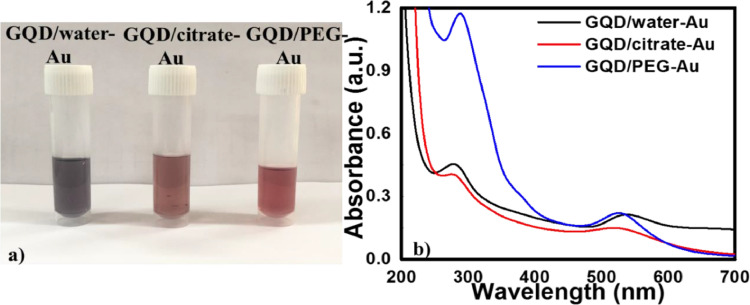
a) Optical images b) UV–Vis absorption spectra of unstabilised and stabilised GQD-Au hybrid particles after 1 week of synthesis.

**Fig. 8:**
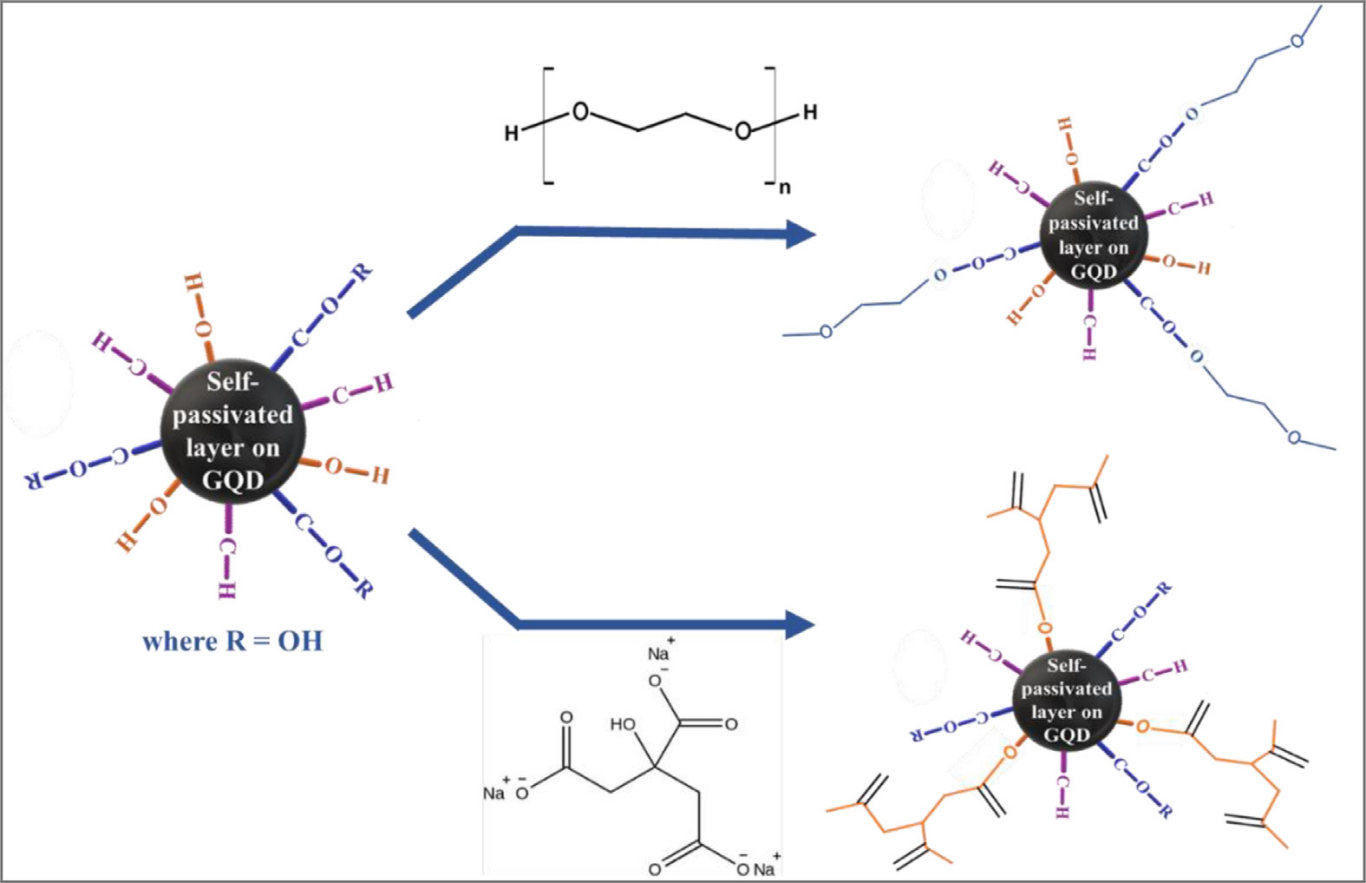
A schematic representation of surface stabilization of GQDs.

## Materials

(i) Reagents

• D-glucose: 0.6 M (Fischer Scientific)
• Trisodium citrate dihydrate: 0.01 M (Fischer Scientific)
• Gold (III) chloride trihydrate: 7 mM (Merck)

(ii) Equipment

• Domestic Microwave (Samsung Combi CEF3JD)
• SEM (Zeiss (MA EVO −18 Special Edition)
• TEM (JEOL/JEM-F200)
• UV–Visible Spectrophotometer (UV 1800 Shimadzu)
• PL (Fluorolog TCSPC Horiba IHR320)
• XRD (Bruker-D8 Discover with Cu-Kα radiation)
• FT-IR (Frontier Perkin Elmer L1280026)
• Dynamic Light Scattering (DLS) (Malvern, v2.0)
• UV-transilluminator (Medox-Bio, MX-1280 – Regular)

(iii) Reagents setup

**0.6** **M D-glucose solution (11.1% solution)** Dissolve 5.6 g of d-glucose in 50 mL of DI water. Store the solution at 10–12 °C (the sample is stable for 1 week under these condition) [15].

**0.01** **M tri-sodium citrate solution** Dissolve 0.15 g of tri-sodium citrate in 50 mL of DI water. Store the solution at 10–12 °C (the sample is stable for 2 weeks under these condition).

**7** **mM Chloroauric acid solution** Dissolve 2 mg of Gold (III) chloride trihydrate in 1 mL of DI water.

## Procedure

**GQD synthesis** ●**TIMING 4 min**

(i) Measure 5 mL of D-glucose solution in a 50 mL - Erlenmeyer flask and place it in a domestic microwave.
(ii) Irradiate the sample at 800 W microwave power for 3–5 min

? TROUBLESHOOT

(iii) The resulting viscous paste is extracted using 5 mL of tri-sodium citrate solution

**! CAUTION** Be careful while removing the Erlenmeyer flask, as it might be hot.

? TROUBLESHOOT

(iv) A pale yellow coloured solution conforms the synthesis of GQDs

**■ PAUSE POINT** Synthesized citrate stabilized GQDs are stable upto 3 weeks when stored at temperature < 4°C.

GQD-Au hybrid nanoparticles synthesis ●TIMING 10 s

(v) To the prepared GQD solution, add 150 µL of HAuCl_4__•_3H_2_O solution. This procedure produces 5 mL of GQD-Au hybrid nanoparticles that are characterized using High Resolution Transmission Electron Microscopy (HR-TEM), UV–Visible (UV–Vis) spectroscopy, X-ray Photoemission Spectroscopy (XPS) and Photoluminescence spectroscopy (PL).

**■ PAUSE POINT** The synthesized citrate stabilized GQD-Au hybrid nanoparticles are stable upto 3 weeks when stored at temperature < 4°C.

? TROUBLESHOOT

**Table:** 

**Step**	**Problem**	**Possible Reason**	**Solution**
(ii)	Alteration in the microwave irradiation time (5 min) results in the formation of dark coloured GQD that does not aid in GQD-Au hybrid nanoparticle synthesis.	The dark coloured GQDs masks the pink coloured appearance of GQD-Au hybrid making the visual identification difficult.	Reduced microwave time (3 min) to obtain a pale yellow coloured solution of GQD, which results in GQD-Au hybrid nanoparticles indicated by cherry red solution.
(iii)	Extraction of the viscous yellow product using DI water results in GQD that are stable up to 1 week at temperatures < 4 °C	The size of GQD increase due to high surface energy resulting in darker solution containing agglomerated particles.	Extraction of the viscous yellow product with stabilizing agent (citrate or PEG) aids in synthesis of stable GQDs and provides a control over the particle size of GQD-Au hybrid nanoparticles

●TIMING

Step 1 and 2, solution preparation and microwave irradiation: 3.5 min
Step 3 and 4, extraction of GQD: 30 s
Step 5, synthesis of GQD-Au hybrid nanoparticles: 10 s

## Method implementation and outputs

### Anticipated results

**Fig. 9:**
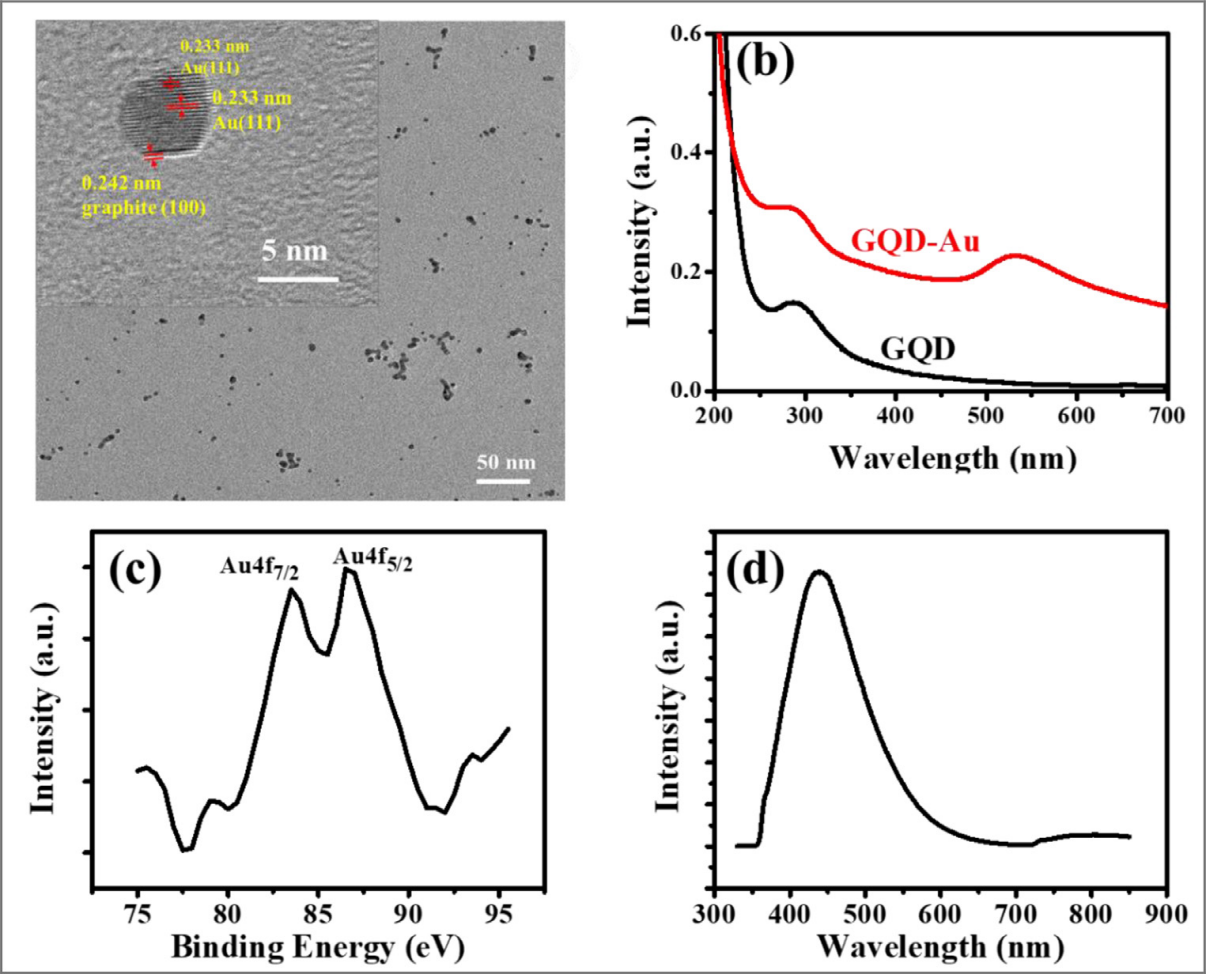
(a) HR-TEM micrograph (b) UV–Vis absorption spectra (c) XPS showing region scan of Au 4*f* region (d) PL emission spectra with excitation wavelength (*λ*_exc_. = 270 nm) of GQD-Au hybrid nanoparticles.

For the first time, a facile and instantaneous protocol has been established for the synthesis of GQD-Au hybrid nanoparticles in the particle size range 5 - 100 nm (Fig. 9a). HR-TEM micrograph (Fig. 9a) also indicates dispersed spherical particle formation. The inset of Fig. 9a exhibits Au (111) lattice fringe distance of 0.233 nm in the inner region and lattice space (100) of graphitic carbon is 0.243 nm on the surface of hybrid nanoparticles [23]. This indicates a core/shell AuNPs@GQD structure formation. Figure S3 further compliments the particle size analysis (see supporting information).Fig. 9b demonstrates absorption peaks at ~ 290 nm in GQD [15] and ~ 286 nm in GQD-Au hybrid. An additional absorption peak at ~ 532 nm is due to Au nanoparticles in GQD-Au hybrid particles. Absorbance peak at ~532 nm is indicative of metallic Au nanoparticles [24]. Face centered cubic crystals of metallic Au nanoparticles is also exhibited by XRD pattern of GQD-Au hybrid nanoparticles (Figure S4, see supporting information). This is further confirmed by the XPS analysis (Fig. 9c) which clearly shows two peaks at binding energy 83.5 and 86.8 eV corresponding to Au 4*f*^7/2^ and Au 4*f*^5/2^ chemical states of Au in GQD-Au hybrid nanoparticles [25]. Fig. 9d shows the emission spectra of GQD and GQD-Au hybrid particles, wherein, the difference in the peak intensity is clearly seen. Chen et al.[23] highlighted that the reduction of chloroauric acid was slightly slower with the GQDs than that with the classic reducing agent sodium borohydride.

**Fig. 10:**
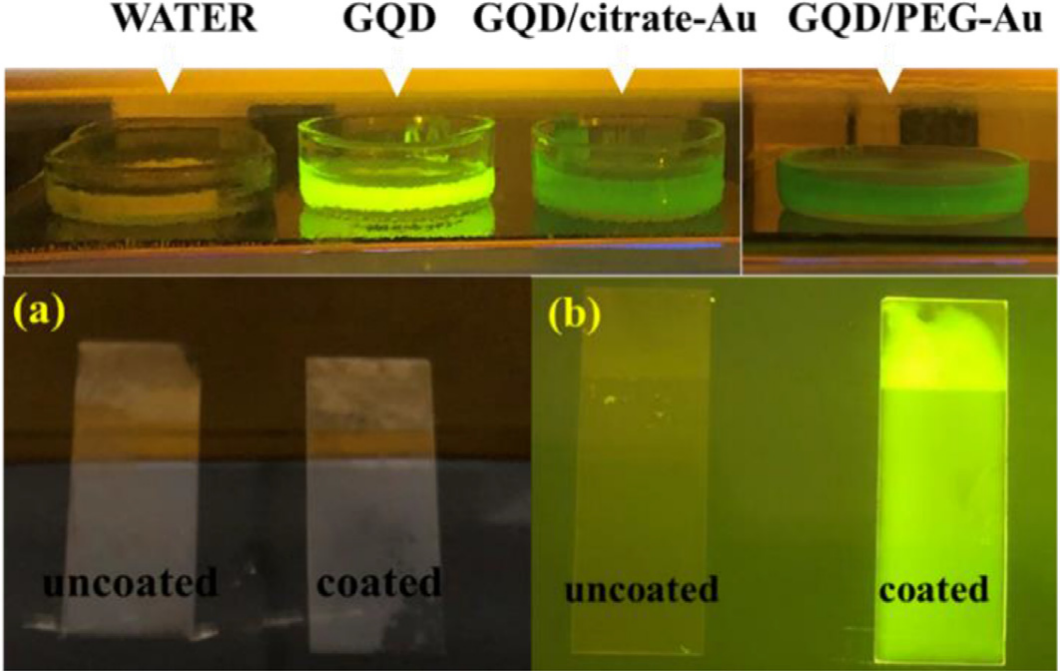
GQD and GQD-Au hybrid particles showing luminescence under UV transilluminator (top). Glass slides (5 x 2 cm^2^) coated with GQD-Au hybrid nanoparticles (a): no light (b): under UV-A illumination.

At first, GQD-Au hybrid nanoparticles were synthesized by extracting the viscous solution of GQD after supplying microwave power of 800 W for 3 min with water, however due to its insufficient stability; stabilizing agents such as citrate helped increase the stability for up to 3 weeks (Fig. 7). From studying various factors such as concentration, volume and pH, it can be concluded that GQD-Au hybrid nanoparticles in the particle size range 5 - 100 nm can be obtained at neutral pH with 7 mM and 30 µL of HAuCl_4__•_3H_2_O (Fig. 5). These GQD-Au hybrid nanoparticles demonstrate luminescence property under UV-A irradiation (Fig. 10), hence, may be used for various applications such as energy-efficient coatings in buildings, as clear films on windows of automobiles/aircrafts *etc*. Synthesized GQD were also used for the reduction of various metal salt such as AgNO_3_ and dyes such as methylene blue (MB) and methyl orange (MO) (Table 1). Absorption peaks at 270 nm and 403 nm exhibit the formation of Ag nanoparticles from its aqueous salt solution (AgNO_3_) in the presence of GQDs after 2 h. GQDs also lead to slow reduction of Methylene blue as indicated by decrease in characteristic absorption peak of Methylene blue at ~ 663 nm. Methyl Orange was completely reduced by GQDs and is indicated by disappearance of absorption peak at ~ 464 nm [26]. Ciganda et al., 2016 [27] indicated that the reducing power of GQDs is similar to Ferrocene.

**Table 1:** Reduction of various metal salts and organic dyes using GQDs.

## Declaration of Competing Interest

The authors declare no competing interests.

## Appendix Supplementary materials

Image, application 1
