# Supplementary material for: Engineering of luminescent graphene quantum dot-gold (GQD-Au) hybrid nanoparticles for functional applications
Source: MethodsX. 2020 Jun 17;7:100963. doi: 10.1016/j.mex.2020.100963 (PMC7327850; doi:10.1016/j.mex.2020.100963)
Supplement: Supplementary file 1 [file mmc1.docx]

*Method article*

**Engineering of luminescent graphene quantum dot-gold (GQD-Au) hybrid nanoparticles for functional applications**

Shikha Wadhwa^a,^*, Alishba T. John^a^, Ashish Mathur^a^, Manika Khanuja^b,*^, Gourav Bhattacharya^c^, Susanta S. Roy^c^, Sekhar C. Ray^d,*^

^a^Amity Institute of Nanotechnology, Amity University Uttar Pradesh, Noida-201313

^b^ Centre for Nanoscience and Nanotechnology, Jamia Millia Islamia, New Delhi-110025

^c^ Shiv Nadar University, Dadri, Gautam Budhha Nagar, Uttar Pradesh-201314

^d^ Department of Physics (CSET), University of South Africa, Private Bag X6, Florida Science Campus, Florida -1710, Christiaan de Wet and Pioneer Avenue, Johannesburg, South Africa.

**SUPPORTING INFORMATION**

**Dynamic Light Scattering (DLS)**


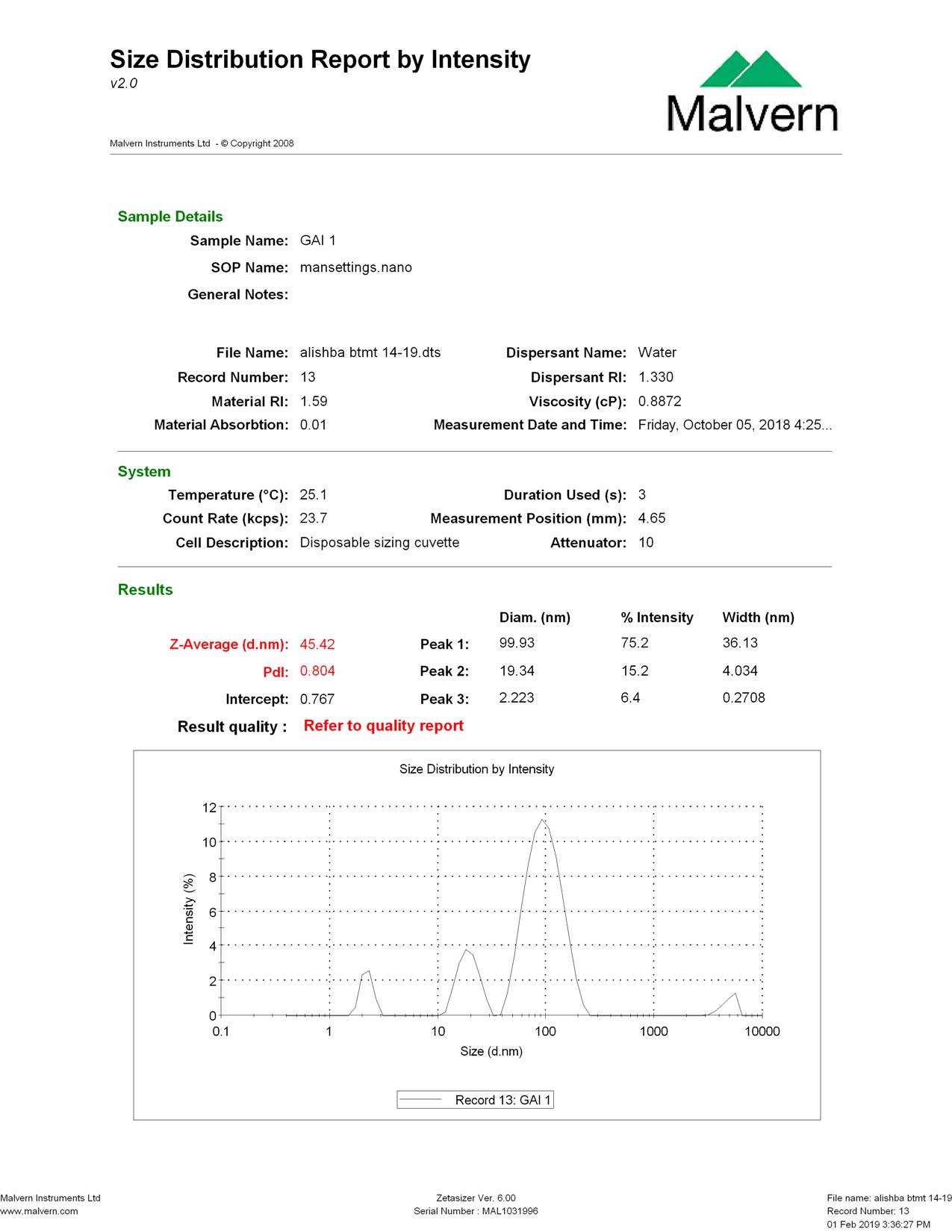


**Figure S1: DLS of GQD-Au hybrid particles**

From the DLS of GQD-Au hybrid particles (Figure S1), it is evident that the particle size is polydisperse with the z-average particle sizes 100 nm and 19 nm, however, merely 15% of the sample contains particles with peak particle size 19 nm. The particle size obtained from HRTEM ranges from 5-100 nm which correlates well with that obtained with DLS. The polydispersity may arise due to some particles which aggregate to form bigger and non-spherical particles. This is also evident from HRTEM images.

**FT-IR analysis**

**Figure S2: FT-IR spectra of GQD and GQD-Au hybrid particles**

The vibrational bands appear at 1028 cm^-1^, 1082 cm^-1^, 1265 cm^-1^ and 1642 cm^-1^ in synthesised GQDs. A vibrational band at 1028 cm^-1^ is due to -C-O bonds in the GQD structure [8]. A vibrational band at 1082 cm^-1^ corresponds to -C-O-C bonds while that at 1642 cm^-1^ is indicative of aromatic bending of C=C bonds and C=O stretches [1-4]. Self-passivation layers on GQD are due to this oxygen containing functional groups on its surface. Vibrational bands observed at 1642 cm^-1^ and 1388 cm^-1^ in GQD-Au synthesised by microwave assisted hydrothermal method are attributed to skeletal vibrations of the aromatic rings of the GQDs.

**Electron Microscopy**

**
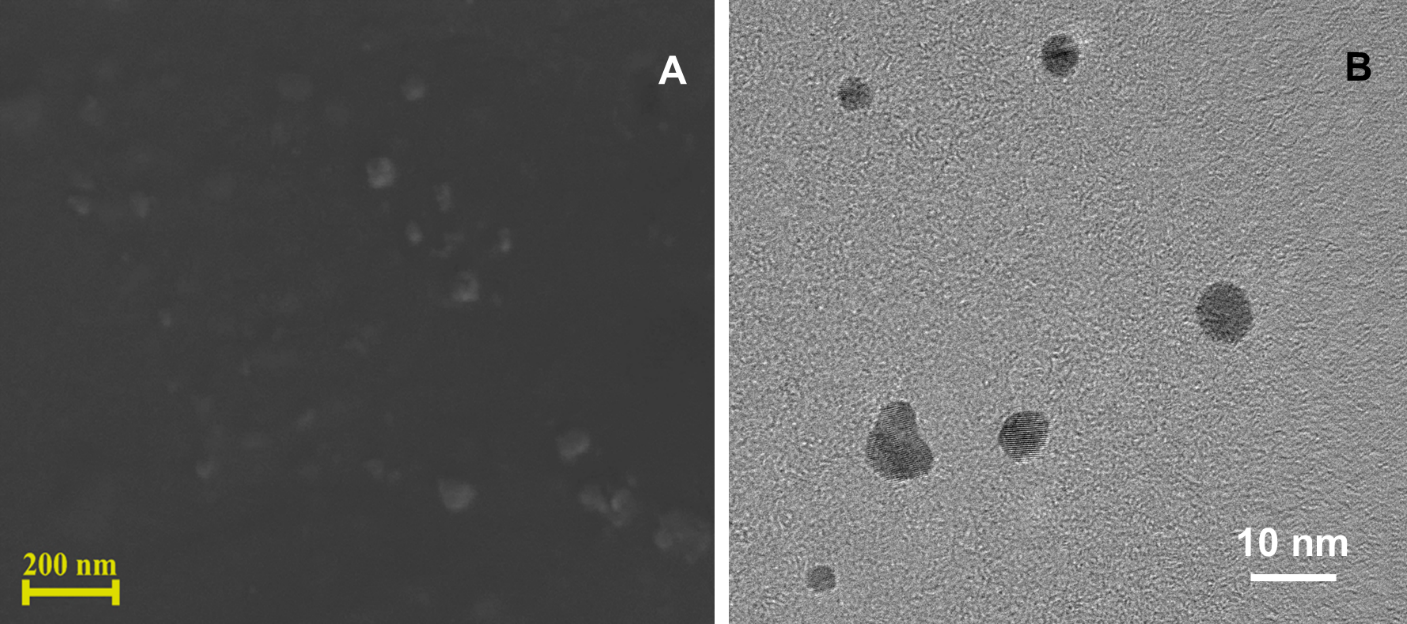
**

**Figure S3: A) SEM image B) HRTEM image of GQD-Au hybrid particles (Magnification: 500 kx)**

SEM image (Figure S3A) exhibits GQD-Au hybrid particles with the particle size of about ~ 100 nm. From HRTEM images (Figure S3 B), uniformly distributed smaller and spherical GQD-Au particles with particle size ~ 5-10 nm can be seen. Addition of tri-sodium citrate stabilised the hybrid particles to a good extent, however, some particles were found to agglomerate and form bigger and non-spherical particles. Hence the sample is polydisperse. This correlates well with the DLS which shows two different particle size ranges.

**XRD Analysis**

**
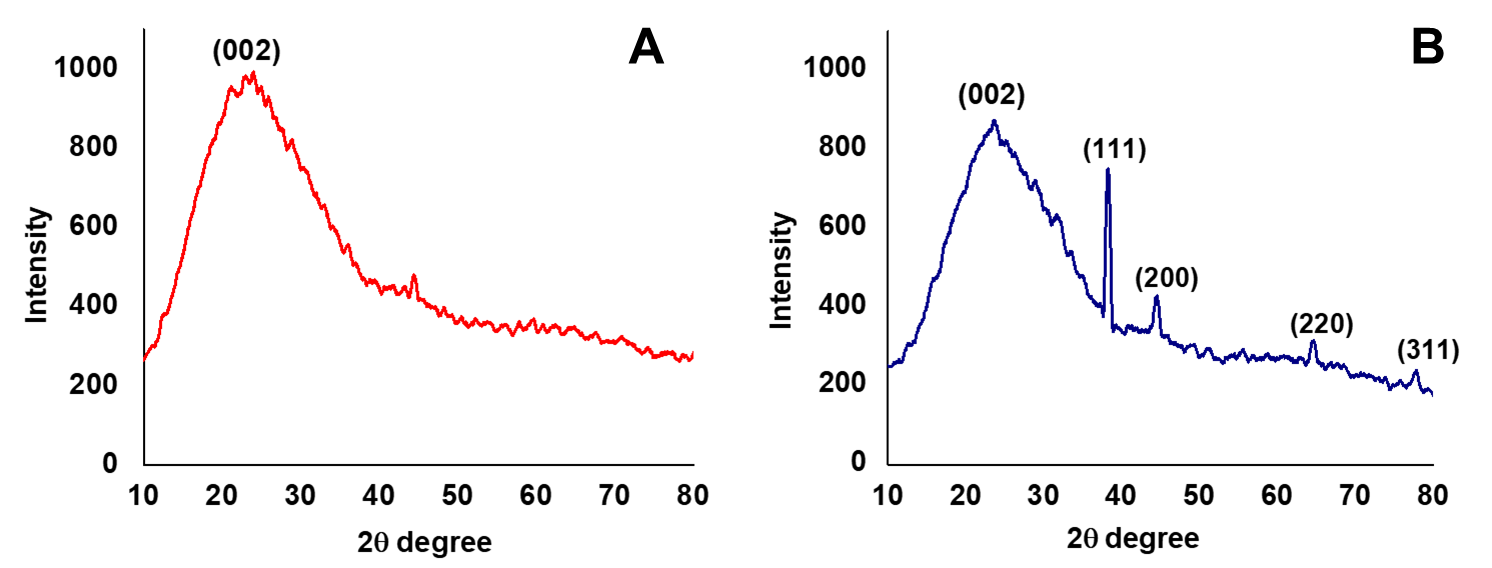
**

**Figure S4: XRD pattern of A) GQDs B) GQD-Au hybrid particles.**

Figure S4 shows a broad XRD peak at 23.40^0^ (002) indicating disordered stacking and thinness of GQDs. In GQD-Au hybrid particles, additional sharp diffraction peaks are obtained at 38.5^0^ (111), 44.3^0^ (200), 64.6^0^ (220) and 78.0^0^ (311) representing face cantered cubic (FCC) lattice of metallic gold nanoparticles [5].

**References**

[1] Choudhary RP, Shukla S, Vaibhav K, Pawar PB, Saxena S. Optical properties of few layered graphene quantum dots. Materials Research Express. 2015 Sep 22;2(9):095024

[2] Li L, Wu G, Yang G, Peng J, Zhao J, Zhu JJ. Focusing on luminescent graphene quantum dots: current status and future perspectives. Nanoscale. 2013;5(10):4015-39

[3] Safajou H, Khojasteh H, Salavati-Niasari M, Mortazavi-Derazkola S. Enhanced photocatalytic degradation of dyes over graphene/Pd/TiO2 nanocomposites: TiO_2_ nanowires versus TiO_2_ nanoparticles. Journal of colloid and interface science. 2017 Jul 15;498:423-32

[4] Mohandes F, Salavati-Niasari M. In vitro comparative study of pure hydroxyapatite nanorods and novel polyethylene glycol/graphene oxide/hydroxyapatite nanocomposite. Journal of nanoparticle research. 2014 Sep 1;16(9):2604.

[5] Wadhwa S, John AT, Nagabooshanam S, Mathur A, Narang J. Graphene quantum dot-gold hybrid nanoparticles integrated aptasensor for ultra-sensitive detection of vitamin D3 towards point-of-care application. Applied Surface Science. 2020 Apr 23:146427
